# Association of HLA-B*51:01, HLA-B*55:01, CYP2C9*3, and Phenytoin-Induced Cutaneous Adverse Drug Reactions in the South Indian Tamil Population

**DOI:** 10.3390/jpm11080737

**Published:** 2021-07-28

**Authors:** Shobana John, Karuppiah Balakrishnan, Chonlaphat Sukasem, Tharmarajan Chinnathambi Vijay Anand, Bhutorn Canyuk, Sutthiporn Pattharachayakul

**Affiliations:** 1 Department of Clinical Pharmacy, Faculty of Pharmaceutical Sciences, Prince of Songkla University, Songkhla, Hat Yai 90110, Thailand; john.shobana@gmail.com; 2 Department of Immunology, School of Biological Sciences, Madurai Kamaraj University, Madurai 625021, Tamil Nadu, India; immunobala@mkuniversity.org; 3 Division of Pharmacogenomics and Personalized Medicine, Department of Pathology, Faculty of Medicine, Ramathibodi Hospital, Mahidol University, Bangkok 10400, Thailand; chonlaphat.suk@mahidol.ac.th; 4 Laboratory for Pharmacogenomics, SomdechPhraDebaratana Medical Center (SDMC), Ramathibodi Hospital, Bangkok 10400, Thailand; 5 Department of Neurology, Meenakshi Mission Hospital and Research Center, Madurai 625107, Tamil Nadu, India; tcvijayanand@gmail.com; 6 Department of Pharmaceutical Chemistry, Faculty of Pharmaceutical Sciences, Prince of Songkla University, Songkhla, Hat Yai 90110, Thailand; cbhutorn@pharmacy.psu.ac.th

**Keywords:** HLA B, CYP2C9*3, cutaneous adverse drug reactions (CADRs), anti-epileptic drugs (AEDS), phenytoin (PHT), genetic risk factors, South India, India

## Abstract

Phenytoin (PHT) is one of the most commonly reported aromatic anti-epileptic drugs (AEDs) to cause cutaneous adverse reactions (CADRs), particularly severe cutaneous adverse reactions (SCARs). Although human leukocyte antigen (HLA)-B*15:02 is associated with PHT-induced Steven Johnson syndrome/toxic epidermal necrosis (SJS/TEN) in East Asians, the association is much weaker than it is reported for carbamazepine (CBZ). In this study, we investigated the association of pharmacogenetic variants of the HLA B gene and CYP2C9*3 with PHT-CADRs in South Indian epileptic patients. This prospective case-controlled study included 25 PHT-induced CADRs, 30 phenytoin-tolerant patients, and 463 (HLA-B) and 82 (CYP2C9*3) normal-controls from previous studies included for the case and normal-control comparison. Six SCARs cases and 19 mild-moderate reactions were observed among the 25 cases. Pooled data analysis was performed for the HLA B*51:01 and PHT-CADRs associations. The Fisher exact test and multivariate binary logistic regression analysis were used to identify the susceptible alleles associated with PHT-CADRs. Multivariate analysis showed that CYP2C9*3 was significantly associated with overall PHT-CADRs (OR = 12.00, 95% CI 2.759–84.87, *p* = 003). In subgroup analysis, CYP2C9*3 and HLA B*55:01 were found to be associated with PHT-SCARs (OR = 12.45, 95% CI 1.138–136.2, *p* = 0.003) and PHT-maculopapular exanthema (MPE) (OR = 4.041, 95% CI 1.125–15.67, *p* = 0.035), respectively. Pooled data analysis has confirmed the association between HLA B*51:01/PHT-SCARs (OR = 6.273, 95% CI 2.24–16.69, *p* = <0.001) and HLA B*51:01/PHT-overall CADRs (OR = 2.323, 95% CI 1.22–5.899, *p* = 0.037). In this study, neither the case nor the control groups had any patients with HLA B*15:02. The risk variables for PHT-SCAR*s,* PHT-overall CADRs, and PHT-MPE were found to be HLA B*51:01, CYP2C9*3, and HLA B*55:01, respectively. These alleles were identified as the risk factors for the first time in the South Indian Tamil population for PHT-CADRs. Further investigation is warranted to establish the clinical relevance of these alleles in this population with larger sample size.

## 1. Introduction

Phenytoin (PHT) is still the most effective treatment for generalized tonic-clonic seizures (GTCS) despite newer anti-epileptic drugs’ (AED) availability [1,2]. However, cutaneous adverse drug reactions (CADRs) may limit its use; the estimated relative risk of PHT-severe cutaneous adverse reactions (SCARs) was reported to be 13% [3]. The prevalence of CADRs ranges from 2–5% in India [4]. Further, PHT and carbamazepine (CBZ) are the worst offenders of CADRs, with incidence rates of 13 and 18%, respectively [5]. The contributing factors to CADRs can be both genetic and non-genetic. In 2004, the association between HLA B*15:02 and CBZ-Steven Johnson syndrome/toxic epidermal necrosis (SJS/TEN) was reported among the Han Chinese populations [6]. Later this association has been confirmed with PHT-SJS/TEN in the Thai and Chinese Asian population [7,8]. As a result, the FDA issued a warning for HLA B*15:02/PHT-SJS/TEN cross-reactivity. However, the strength of this association is weaker than CBZ-SJS/TEN and not demonstrated well enough in many populations [9,10].

Polymorphisms in genes that encode drug-metabolizing enzymes, in addition to HLA-B alleles, play a key role in the initiation of CADRs by slowing drug metabolism. PHT is metabolized primarily by CYP 450 in phase I reactions and mostly by UDP-glucuronosyl transferase (UGT) in phase II reactions. The enzymes *CYP2C9* and *CYP2C19* are responsible for 90% and 10% of the metabolism of PHT, respectively [11].In CYP2C9, 2* and 3* variants are responsible for reduced PHT clearance [12,13].

Due to distinct waves of immigration, dissimilar genetic patterns in the Indian population have been extensively documented: North Indians are genetically closer to Caucasians, whilst Central Indians are closer to Asians than the European group [14,15]. The South Indians, or Dravidians, who are distributed on the southern side of India (peninsular India), are the original inhabitants of the Indian sub-continent; thus, the distribution of polymorphic alleles is not homogenous [16,17]. For example, the frequency of HLA B*15:02 is lower (0%) among West Coast Parsi and higher (6%) among Pawra in the Khandesh region [18,19]. Similarly, the frequency of CYP2C9*3 varies by location in India: a higher frequency (7–9%) is seen in the Dravidian population, whereas the prevalence is relatively low (0–2%) in the North Indian population [20].

A few studies in India have reported the association of HLA alleles and AEDs induced CADRs [21,22,23,24], and one study from South India reported a high serum PHT concentration in healthy volunteers carrying CYP2C9*3 [19]. There has been little research into the genetic risk factors for PHT-CADRs in the South Indian Tamils, who inhabit primarily in Tamil Nadu, India, and also scantily in Malaysia, Sri Lanka, Singapore, and Mauritius. We aimed to look at the relationship between HLA-B alleles, CYP2C9*3, and PHT-CADRs in the South Indian Tamil population for the first time in this study.

## 2. Methodology

### 2.1. Study Design and Settings

**Figure 1:**
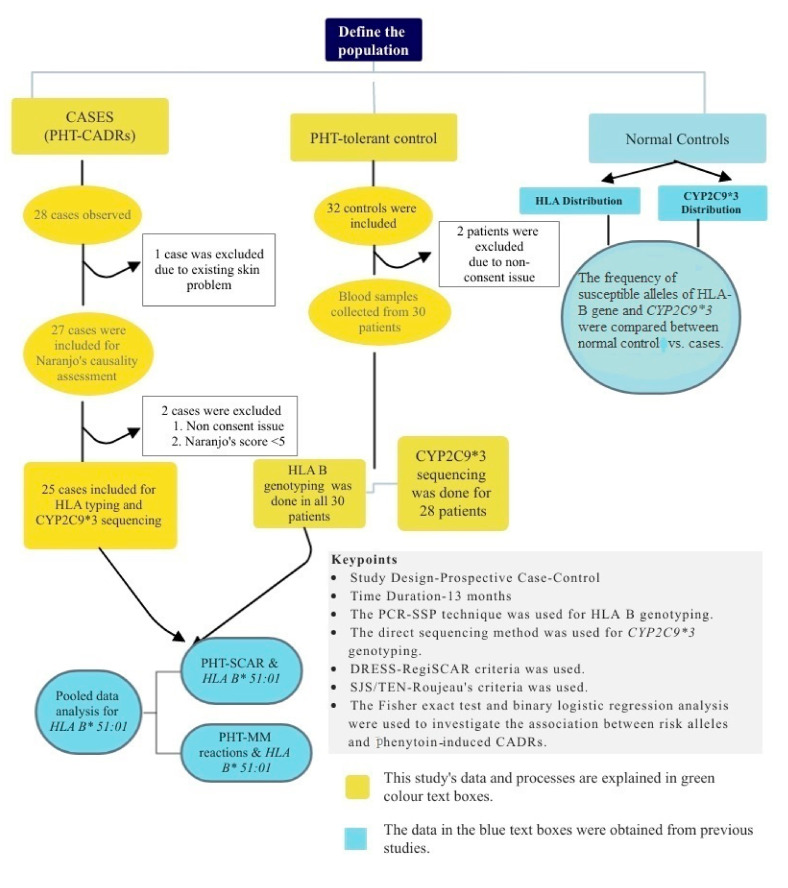
Flowchart of study design.

This study was conducted as a prospective case-controlled study over a period of 13-months in the Neurology-Outpatient Department (OPD), Neurology ward, and Neurology-Intensive Care Unit (ICU) of Meenakshi Mission Hospital and Research Center (MMHRC) in Madurai, Tamil Nadu, India. This study was approved by the Institutional Ethics Committee Board of MMHRC (MMHRC/IEC/07/2018). DNA analysis of both cases and tolerant controls was performed at the Immunology Department, School of Biological Sciences, Madurai Kamaraj University, Madurai, Tamil Nadu, India (Figure 1).

### 2.2. Participants

Patients who had reported CADRs within 12 weeks of using PHT were included in the study. Patients on PHT for more than 3 months and had no signs or symptoms of CADRs were considered tolerant controls. The patients who signed the consent form and agreed to give 3 mL of whole blood were included. Patients experiencing other PHT-ADRs and with established skin problems, such as psoriasis and contact dermatitis, were eliminated. Normal-control data was retrieved only from South Indian populations, but no such restrictions were kept for pooled data analysis.

The dermatologist at MMHRC diagnosed all of the PHT-CADRs as well as additional diagnostic criteria, such as the temporal relationship with phenytoin, clinical morphology of the skin, and mucosal and systemic involvement. SJS/TEN, acute generalized exanthematous pustulosis (AGEP), exfoliative dermatitis (ED), drug rash with eosinophilia and systemic symptoms (DRESS) reactions were considered as SCARs. The remaining reactions with no/less mucosal involvement were considered to be mild-moderate reactions in this study [25]. Maculopapular exanthema was defined as a rash characterized by fine pink macules/papules/lesions on the skin with no mucosal or systemic involvement (MPE) [26]. Acneiform drug eruption was defined as a monomorphic eruption without comedones (AFDE) [27].

Fixed drug eruption (FDE) was defined as a single round and oral, sharply demarcated, red-lined lesion with a diameter of 1–10 cm [28]. PHT-lichenoid drug eruption was defined as skin lesions characterized by scaling and hypertrophic pigmentation, generally in combination with oral eruption (LDE) [29]. Patients with rapidly developing dark purpuric macules, atypical target lesions, blisters accompanied by mucosal and skin detachment were diagnosed with SJS, according to Roujeau’s criteria [30]. The RegiSCAR criteria were used to diagnose DRESS and DHS, which included an acute skin rash with at least one internal organ lymphadenopathy; hematologic abnormalities, such as eosinophilia and atypical lymphocytosis; and fever [25]. Patients with erythematic inflammatory skin disease, scaling on the cutaneous surface of the skin, thickened skin, itching, swollen lymph nodes, fever, and fluid loss were considered as PHT-ED [31].

Once the patient was identified as a case or control, the consent form was handed to them along with a patient information document created in the local language of Tamil. The normal-control data for the HLA-B association study was obtained from Leenam Dedhia et al. (2015), who investigated HLA diversity and its significance in South Indians [32]. The normal control data for CYP2C9*3 and PHT-CADRs association testing is from the published literature (Nahar R et al. 2013) [33].

### 2.3. Causality Assessment

The causality of PHT-CADRs was assessed using Naranjo’s scale, with patients scoring 9 (definite) and 5–8 (probable) included in this study [34]. Patients reported with DRESS were included if the RegiSCAR score was definite >5 and probable (4–5) [35]. The ALDEN score was used to assess the drug’s causality with SJS: patients scoring very probable >6 or probable 4–5 were included [36].

### 2.4. Genotyping

#### 2.4.1. DNA Extraction/HLA-B Genotyping

DNA was extracted from 3mL of peripheral blood using the salting-out method [37,38]. A UV spectrophotometer was used to measure the concentration and purity of DNA by measuring its optical density (OD) at 260 nm. The polymerase chain reactions-sequence-specific primer technique was used to genotype HLA-B. (PCR-SSP) (Applied Biosystems Verti-Thermal cycler, Thermo Fisher Scientific, Waltham, MA, USA) [39].

#### 2.4.2. CYP2C9*3 Sequencing

The reference DNA sequence of the target variant CYP2C9*3 was retrieved (rs1057910), and the genomic DNA was amplified using the selected forward primer from chromosome passion at 94981018 to 94981037 bp (GTGCATCTGTAACCATCCTC) and the reverse complementary primer from chromosome passion at 94981455 to 94981476 bp (GAGTTATGCACTTCTCTCACCC). The PCR DNA was purified according to the manufacturer’s protocol by MinElute PCR Purification Kit (Cat. No. 28006, Qiagen, Valencia, CA, USA) and sequenced using a 3500 automatic DNA segmentation analyser (3730 DNA analyzer, Applied Biosystems, Thermo Fisher Scientific, Waltham, MA, USA). Sequence scanner software was used to obtain sequential sequencing.

### 2.5. Statistical Analysis

To compare the demographics and clinical features of the case and control groups, a Student’s *t*-test was performed. The Fisher exact or Pearson chi-square tests were used if the demographics are categorical variables. The OR was calculated to see if there was an association between specific pharmacogenetic risk factors and PHT-CADRs. The Fisher exact test was used to get the *p*-value. Woolf’s logit method was used for any cells in a contingency table that had zero. The bivariate analysis was carried out on alleles with a prevalence of more than 5% in the case group. To examine the association of various risk alleles with PHT-induced CADRs, PGx variants that exhibit significant association in bivariate analysis were included in a multivariate binary logistic regression analysis.

We used pooled data analysis for the HLA B*51:01 to boost the study’s power. The HLA B*51:01 case and tolerant control data were obtained from one of the North Indian studies that reported the relationship between HLA alleles and AED-induced CADRs [20].

All the statistical analysis was performed using GraphPad Prism 8. After Bonferroni correction, a *p*-value 0.008 (<0.05/6—two-tailed) was considered significant.

## 3. Results

### 3.1. Patient Demographics

This study included 30 PHT-tolerant and 25 PHT-induced CADRs epileptic cases in this study. Six (23.07%) of the 25 cases were SCARs, including two cases of SJS, three cases of DRESS, and one case of ED. The remaining 19 cases (76.92%) were mild-moderate reactions, comprised of 15 MPE, 2 AFDE, 1 LDE, and 1 FDE. The case group included 14 males and 11 females with a mean age of 40.60 ± 18.15 years, while the PHT-tolerant group included 18 males and 12 females with a mean age of 36.21 ± 14.71 years. Epilepsy, seizure, cerebrovascular accidents, and CNS infections were the common indications for PHT in both the case and tolerant groups (Table 1) (Supplementary Table S1).

The CYP2C9 normal-control data from the previous study included 82 healthy people (40 males and 42 females) from Dravidian or South Indian populations, such as Tamils (25), Andhra Pradesh (32), and Kerala [35]. This study included the data of 463 HLA-B normal-controls (Tamils) that was retrieved from past literature [34]. A total of 52 PHT-CADRs patients (31 MPE, 7 SJS/TEN, 8 DRESS, and 5 FDE) and 100 PHT-tolerant control patients with ages ranging from 6 to 72 were included in the pooled analysis. There were 22/30 and 41/59 females and males in their case and tolerant groups, respectively [20].

### 3.2. Clinical Features of PHT-CADRs

The itching was more common among mild-moderate reactions (13/20) than SCARs (1/6). Maculopapular rash/exanthematous rash or lesions/skinredness/burning sensation or warmth while touching were the most common cutaneous clinical manifestations in mild-moderate reactions (6/6 in SCARs and 13/20). Papules, pustules, blisters, and erythema were other serious features of skin reactions that were more common with SCARs (4/6) than mild to moderate reactions (5/20). The most commonly impacted mucosal sites were the mouth, eyes, genitals, and anogenital mucosa. SCARs were the only ones that showed systemic involvement. The most commonly affected systems were the liver and hematological systems. All three DRESS patients showed lymphadenopathy, abnormal lymphocytes, and eosinophilia. In one ED patient, neutrophilic leukocytosis was observed (Table 1).

The onset latency period ranged from 7 to 42 days, with a mean of 21.7 days. In Naranjo’s causation assessment, all cases received a likely score of ≥5. According to the RegiSCAR and ALDEN criteria, all DRESS and SJS patients had a definite (>5) and very probable (>6) connection.

### 3.3. Frequency of HLA-B Alleles in PHT-Cases and Tolerant Controls

**Figure 2:**
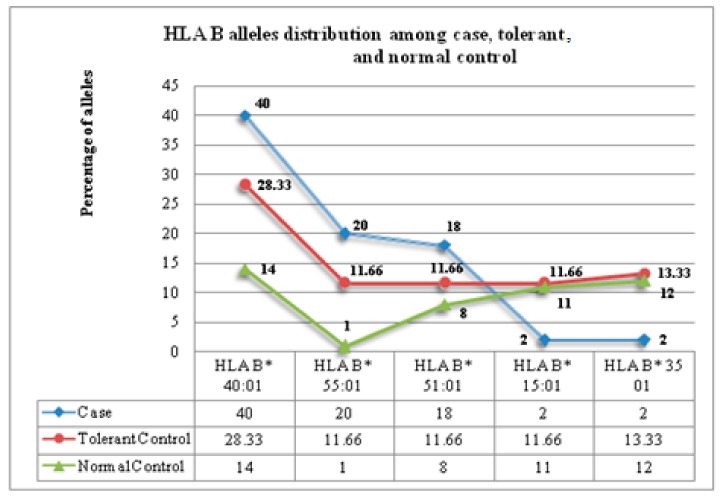
Frequently observed HLA B alleles among PHT-CADRs and PHT-tolerant controls.

HLA-B genotyping data from PHT-CADRs cases showed higher frequencies (>5%) of HLA B*40:01 (40%), HLA B*55:01 (20%), HLA B*51:01 (18%), and HLA B*07:02 (10%) alleles and lower frequencies (<5) of HLA B*57:01 (4%), HLA B*52:01(2%), HLA B*15:01 (2%), HLA B*07:01, and HLA B*35:01(2%).In tolerant-controls, the following alleles were observed more frequently (>5%): HLA B*40:01 (28.33%), HLA B*55:01 (11.66%), HLA B*51:01 (11.66%), and HLA B*07:02 (6.66%), HLA B*52:01(6.66%), HLA B*15:01 (11.66%), and HLA B*35:01 (13.33%). On the other hand, HLA B*07:01 and HLA B*54:01 were reported at lower frequencies (Figure 2 and Supplementary Table S1).

### 3.4. Bivariate Analysis of HLA B Alleles and PHT-CADRs

#### 3.4.1. SCARs

All six SCARs patients carried HLA B*51:01 allele against 7 out of 30 PHT-tolerant controls. In sub-group analysis, the association between PHT-DRESS was well demonstrated with both tolerant (OR = 21.93, 95% CI 1.013–474.9, *p* = 0.022) and normal controls (OR = 42.54, 95% CI 2.114–855.8, 0.003). The association between PHT-SJS and HLA B*51:01 was marginal with the PHT-tolerant control group (*p* = 0.072) and stronger with normal control group (*p* = 0.021), respectively. HLA B*40:01 was reported in all DRESS (3) and ED (1) cases against 14 out of 30 tolerant patients (28.33%), three of which were found to be homozygous. The association between PHT/DRESS and HLA B*40:01 was insignificant as the comparison made with PHT-tolerant group (*p* = 0.227) but found to be significant as the cases compared with normal control group (*p* = 0.0001). In this study, no other HLA B alleles were identified to be related to PHT-SCARs. The HLA B*51:01 genetic marker to PHT-SCAR has a significant positive/negative predictive value (46/100).

#### 3.4.2. Mild-Moderate Reactions

The HLA B*40:01 allele was detected in 14 patients (73.68%) in the mild-moderate reaction group, and two PHT-MPE patients were homozygous for it. When compared to normal control, the association between HLA B*40:01/PHT-mild-moderate reactions, particularly MPE (OR = 10.45, 95% CI 3.299–29.34, *p* = 0.0001) and AFDE (OR = 25.65, 95% CI 1.172–552.8, *p* = 0.028), was found to be significant. The HLA B*55:01 allele was the second most common among the mild-moderate reaction group. Only PHT-MPE patients had a greater frequency of this allele (33.33%), and homozygosity was found in one patient. The association between HLA B*55:01 and PHT/MPE was stronger in both PHT-tolerant (OR = 4.929, 95% CI 1.322–17.33, *p* = 0.022*) and normal control group (OR = 204.0, 95% CI 27.72–2229, *p* < 0.0001*) with high positive/negative predictive value (56/79).

#### 3.4.3. Overall PHT-Induced CADRs

HLA B*51:01, HLA B*40:01, and HLA B*55:01 alleles were shown to have a significant association with overall PHT-CADRs when compared to normal controls, (HLA B*51:01; OR = 3.43, 95% CI 1.422–8.652, *p* = 0.017; HLA B*40:01, OR = 13.44, 95% CI 4.849–33.14, *p* = <0.0001; HLA B*55:01;OR = 76.50, 95% CI 10.40–841.9, *p* < 0.0001), but none of them were found to have a significant association with PHT-overall CADRs when compared to PHT-tolerant controls. The PHT-tolerant group showed higher frequencies of HLA B*15:01 and HLA B*35:01 than the case and normal control groups. For these alleles, the negative association with PHT-CADRs was found to be significant. (HLA B*15:01; OR = 0.1369, 95% CI of 0.0117–0.9204, *p* = 0.0593 and HLA B*35:01; OR = 0.1146, 95% CI 0.0099–0.7273, *p* = 0.0307) (Table 2).

#### 3.4.4. CYP2C9*3

In the current study, the CYP2C9*3 (AC) genotype was found in 12 out of 25 PHT-CADRs cases, but only two patients in the PHT-tolerant group had this allele, and the homozygous (CC) genotype was not found in either case or the tolerant control group (Table 3). The association testing was performed between case vs. PHT-tolerant and cases vs normal-control [23]. In both comparisons, the analysis revealed a stronger association between CYP2C9*3 and overall PHT-CADRs (case vs. PHT-tolerant; OR = 12.92: 95% CI 2.777–61.46, *p* = 0.0006, case vs. normal control; OR = 5.385, 95% CI 1.917–13.67 *p* = 0.0017). SCARs (OR = 26.00, 95% CI2.855–1720, *p* = 0.0043) and mild-moderate reactions (OR = 9.455; 95% CI1.628–47.25, *p* = 0.0086) both showed a positive susceptibility association with CYP2C9*3 in sub-group analysis. All three DRESS patients had this mutant allele in SCARs, and the relationship was found to be significant (OR = 74.20, 95% CI 2.922–1884, *p* = 0.002). This allele was observed in 5 MPE, 1 AFDE, 1 LDE, and 1 FDE patients with mild-moderate reactions. The MPE was found to have a substantial association withCYP2C9*3 (OR = 6.500, 95% CI 1.008–35.01, *p* = 0.039) (Table 2).

### 3.5. Pooled Data Analysis

Prior to pooling, the association between HLA B*51:01 and PHT-SCARs was shown to be stronger with broad CI (OR = 40.73, 95% CI 2.045–811.3, *p* = 0.0009) but not with overall PHT-CADRs (OR = 0.848, 95% CI 0.5871–0.377, *p* = 0.377).The number of CADRs increased to 75 after pooling, with 21 patients carrying HLA B*51:01, while the number of tolerant controls climbed to 130, with 15 HLA B*51:01 carriers.HLA B*51:01 and PHT-overall CADRs were found to have a significant relationship (OR = 2.323, 95% CI1.122–5.899, *p* = 0.037). Even after pooling the data, the connection between HLA B* 51:01 and mild-moderate reactions is weaker (*p* = 0.07) (Table 3).

### 3.6. Multivariate Binary Logistic Regression Analysis

The multivariate binary logistic regression analysis showed a stronger association between CYP2C9*3 and PHT-induced all kinds of CADRs, and it was the only predictor that was related to both severe and mild-moderate reactions (OR = 4.041, 95% CI 1.125–15.6, *p* = 0.035). In subgroup analysis, PHT-MPE was shown to be highly associated with HLA B*55:01 (OR = 12.00, 95%CI 2.759–84.82, *p* = 0.003), and CYP2C9*3 was the sole predictor variable with a significant association with PHT-SCARs (OR = 12.00, 95%CI 2.759–84.82, *p* = 0.003) (Table 4).

## 4. Discussion

In Asian populations, HLA B*51:01 has been related to PHT-induced CADRs, particularly with PHT-SCARs [40,41].A recent multicenter East Asian study that investigated the genetic predictors of PHT-hypersensitivity reported that concurrent testing of HLA B* 13:01/HLA B* 15:02/HLA B*51:01 and CYP2C9*3 would help in identifying individuals at risk of developing PHT-CADRs [42]. In India, one of the North Indian studies reported HLA B*57:01 (OR = 11.00, 95% CI: 1.41–85.81, *p* = 0.05) and HLA B*51:01 (OR = 6.90, 95% CI: 1.38–34.29, *p* = 0.007) as risk factors for CBZ-SJS and PHT-DRESS, respectively [20]. In the current study, the HLA B*51:01 allele was found to be strongly associated with PHT-SCARs, especially DRESS, and the pooled data analysis corroborated this relationship with both SCARs and overall CADRs. However, this allele was presented in all of our SCAR patients (6/6), whereas only 4 of 15 patients in pooled data had this allele, which could be attributable to distribution variations. In Mumbai (central India), for example, it is more frequent among Patels (19.60%) and Iyers (17.60%), whereas it is less common among Marathas (4.84%) [43] and North Indian Hindus (3.5%) (Lucknow) [44]. Its distribution among South Indians, particularly Tamils, ranges between 8–12.5%.

A study from South India reported that HLA B*07 was the most common allele (6–13%) in the HLA-B gene, and its association with cervical cancer along with *HLA DQ8* was found to be significant. The next common alleles reported in South Indians were the split antigens of the HLA B5 serotype, HLA B* 51 (8–12.5%) and HLA B* 52 (5–10%). Their association with different vasculitides was reported (HLA B*51 and Behchet’sdiseases, HLA B* 52, and Takayasu’s arteritis) [45].

In this study, the association between HLA B*40:01 and PHT-mild moderate reactions in particular, MPE, was found to be stronger when compared to normal healthy controls, confirming previous findings (Sukesm et al., 2020) [46], which confirmed HLA B*40:01 as a risk factor for PHT-induced MPE (OR 3.647; 95% CI, 1.193–11.147; *p* = 0.023).This allele could be a drug-specific HLA genetic marker for PHT-MPE. However, a study with a larger cohort is needed to confirm this finding.

HLA B*55:01 was not shown to be susceptible to PHT-induced CADRs in any previous association studies that looked into the relationship between HLA and AEDs. The current study is the first to confirm this association in the South Indian-Tamil population. However, a Han-Chinese study also showed a correlation between HLA B*55:01 and LTG-induced MPE (OR = 24.78, 95% CI 1.50–408.76, *p* = 0.08) [47]. This genotype has been linked to penicillin hypersensitivity and nevirapine-induced SCARs in addition to AEDs [48,49]. In this study, HLA B*15:01 and HLA B*35:01 were found in a higher percentage of PHT-tolerant people than in cases and normal controls. This finding is consistent with other Indian studies that indicated a higher prevalence of the HLA B*15:01 allele in the control group than in the case group [23,24], whereas HLA B*35:01 was associated with LTG-induced MPE [50]. In the Tamil population, HLA B*15:01 and HLA B*35:01 may be protective alleles for PHT-CADRs.

The mutant allele (AC) was present in 48 and 7.14 percent of cases and tolerant-control groups, respectively, with no homozygosity (CC), which is similar to a study that found no frequency of CC genotype in the South Indian Dravidian community [19]. The current investigation found a substantial link between the CYP2C9*3 heterozygous condition and PHT-induced CADRs. In this investigation, patients with CYP2C9*3 alleles were almost 13 times more vulnerable to PHT-CADRs than the tolerant group. This finding is comparable to that of a Thai study, which found that patients with CYP2C9*3 have a 14.5 times higher incidence of PHT-SCARs. A subgroup analysis of this study within the SCARs group reveals a higher correlation between CYP2C9*3 and PHT-induced DRESS, which was also corroborated in another Thai investigation [46,51,52].

There may be some limitations to this research. We did not rule out patients who were using CYP2C9 inhibitors, which could have contributed to the rise in PHT levels. Despite this, only three patients were prescribed VPA (CYP2C9 inhibitor), and no other known CYP2C9 inhibitors were prescribed in this group. Although a few studies have found a relationship between CYP2C9*2 and PHT-CADRs, we did not investigate this allele in this study because it is extremely rare (1–2%) in the South Indian population. Another limitation of our research is the small sample size. The rare outcome of interest is the reason for it. In addition to genetic defects, clinical and non-clinical factors may play a role in the initiation of PHT-CADRs, and these should be examined alongside genetic variants.

## 5. Conclusions

CYP2C9*3 and HLA B*51:01 were found to be associated with PHT-SCARs and PHT-DRESS. On the other hand, PHT-mild/moderate cutaneous reactions are linked with HLA B*55:01 and HLA B*40:01 in this study. This is the first study in South India, specifically among Tamils, to show a correlation between HLA B*51:01, HLA B*55:01, and CYP2C9*3 alleles and PHT-CADRs. These alleles can be employed as genetic markers to identify individuals who are susceptible to PHT-CADRs and to ensure that PHT is as safe as possible for Tamil epileptic patients. Furthermore, our findings highlight the necessity of including the HLA B*5101 and CYP2C9*3 alleles into a pre-emptive genetic testing panel for Asians with PHT-CADRs.

## Figures and Tables

**Table 1:** Patient demographics and clinical manifestations of PHT-CADRs and PHT-tolerant group.

Parameters		PHT-Induced CADRsN (%)		Total (25)	Tolerant (30)	*p* Value
		Severe (6)	MPE and other Mild-Moderate Reactions ^c^ (19)			
Age	<20	02 (33.33)	1 (5.26)	3 (12)	5 (16.66)	0.71
	21–40	01 (16.66)	8 (42.10)	9 (36)	11 (36.66)	0.80
	41–60	2 (33.33)	6 (31.57)	8 (32)	12 (40)	0.26
	>60	2 (33.33)	3 (15.78)	5 (20)	02 (6.66)	0.12
Mean age ± SD		44.5 ± 21.77	39.75 ± 1.414	42.15 ± 3.35	36.21 ± 14.71	0.36 ^a^
No of comorbidities (Mean)		2 ± 0.00	1 ± 1.05	1.54 ± 0.82	037 ± 0.75	0.01
Gender	Male	04 (66.66)	10 (52.63)	14 (56)	18 (60)	0.59 ^b^
	Female	02 (33.33)	09	11 (44)	12 (40)	
Onset latency (days)		9–26	7–42	7–42	-	-
Indications	Seizure	3 (50)	9 (47.36)	12 (48)	11 (36.66)	-
	Epilepsy	2 (33.33)	8 (42.10)	10 (40)	16 (53.33)	-
	CVA	1 (16.66)	1 (5.26)	02 (8)	03 (10)	-
	Others	0	1 (5.26)	01 (4)	-	-
Social History	Alcohol (Yes)	1 (16.66)	6 (31.57)	7 (28)	8 (26.66)	>0.99
	Smoking (Yes)	0	3 (15.78)	3 (12)	1 (3.33)	0.31
Causality scores	Naranjo’s	7	5–7	5–7	-	-
	RegiSCARS	>5	-	>5	-	-
	ALDEN	7–8		7–8	-	-
CM/Skin	Itching	1 (16.66)	13 (68.42)	14 (56)	-	-
	Pap. Rash/Redness	6 (100)	19 (100)	25 (25)	-	-
	Papules/Pustules	4 (66.66)	04 (21.05)	08 (32)	-	-
	Blistering/Peeling	2 (33.33)	0	02 (8)	-	-
	Erythema P/P	3 (50)	0	03 (12)	-	-
CM/Mucosa	Eye	6 (100)	2 (10.52)	08 (32)	-	-
	Oral Mucosa	6 (100)	0	06 (24)	-	-
	Genital	4 (66.66)	0	04 (16)	-	-
	Anogenital	2 (33.33)	0	02 (8)	-	-
	F.Edema	4 (66.66)	2 (10.52)	06 (24)	-	-
CM/Systemic abnormalities	Fever	6 (100)	3 (10.52)	09 (36)	-	-
Liver						
AST IU/L (Mean ± SD)	SJS/DRESS/ED	96.50 ± 9.192/183.7 ± 96.66/185	-	-	-	-
ALT IU/L(Mean ± SD)	SJS/DRESS/ED	72.00 ± 12.73/241.3 ± 139.9/131	-	-	-	-
Alk.Phosphatase	SJS/DRESS/ED	117.5 ± 17.68/331.0 ± 128.7/401	-	-	-	-
Haematological abn.						
WBC (Mean ± SD)	SJS/DRESS/ED	5200 ± 424.3/11200 ± 2458/8000/µL	-	-	-	-
Lymphocytes (M %)	SJS/DRESS/ED	17%/56%/-	-	-	-	-
Eosinophil (M %)	SJS/DRESS/ED	09/12/-	-	-	-	-
Lymphadnopathy	SJS/DRESS/ED	Absent/3/Absent	-	-	-	-
AEDs Combination	PHT+VPA	2 (33.33)	2 (10.00)	4 (15.38)	6 (18.75)	>0.999
	PHT+LEV	2 (33.33)	5 (25.00)	7 (26.92)	4 (12.50)	0.1935
	PHT+VPA+LEV	-	1 (5.00)	1 (03.84)	-	0.4576
	Others	2 (33.33)	2 (10.00)	4 (15.38)	0	-

SJS, Steven Johnson syndrome; DRESS, drug reactions eosinophilia systemic syndrome; ED, exfoliative dermatitis; MPR, maculo-papular rash; ^c^ FDE, fixed drug eruption; LDE, lichenoid drug eruption; AFDE, acneiform drug eruption were in others category, ^a^ *p* value was calculated by independent Student’s *t* test, ^b^ *p*-value calculated by Fisher’s exact test.

**Table 2:** Bivariate analyses of association between HLA B alleles and CYP2C9*3 with PHT-CADRs.

HLA B Alleles	Phenotype (no of Cases)	Number of Cases			Case vs. Tolerant		Case vs. Healthy Population		PPV/NPV	Sensitivity/Specificity
		Case *n* = 25	Tolerant *n* = 30^HLA B^/28^CYP2C9*3^	General Population ^R1 & R2^ *n* = 463/82	OR (95% CI)	*p* Value ^a^	OR (95% CI)	*p* Value ^a^		
		N (%)	N (%)	N (%)						
PHT-SCARs										
B*51/51: 01	SJS (2)	2 (100)	7 (23.33)	19 (13.86)	15.67 (0.6743–64.0)	0.07	30.38 (1.405–657.1)	0.02	22/100	100/76
	DRESS (3)	3 (100)			21.93 (1.013–474.9)	0.02	42.54 (2.114–855.8)	0.003	30/100	100/76
	ED (1)	1 (100)			9.40 (0.3452–56.0)	0.25	18.23 (0.716–463.8)	0.14	12.5/100	100/76
B* 40/40:01	DRESS (3)	3 (100)	14 (46.66)	22 (16.05)	7.96 (0.3787–67.5)	0.22	35.93 (1.794–719.9)	0.005	17/100	100/53
	ED (1)	1 (100)			3.41 (0.1288–0.49)	0.48	15.40 (0.607–390.2)	0.16	6.6/100	100/53
B* 5101	SCARs (6)	6 (100)	7 (23.33)	19 (13.86)	40.73 (2.045–811.3)	0.0009	296.3 (16.11–5450)	<0.001	46/100	100/76
B* 4001	SCARs (6)	4 (66.66)	14 (46.66)	22 (16.05)	6.571 (1.157–37.69)	0.057	40.09 (8.70–213.6)	0.0001	36/92	66/76
PHT-Mild-Moderate										
B* 40/40:01	MPE 15)	10 (40)	14 (28.33)	22 (16.05)	2.286( 0.6742–7.887)	0.342	10.45 (3.299–29.34)	<0.001	41/76	66/53
	AFDE (2)	2 (50)			5.690 (0.2519–128.5)	0.483	25.65 (1.172–552.8)	0.02	12.5/100	100/53
	LDE (1)	1 (50)			3.414 (0.1288–90.49)	0.483	15.40 (0.607–390.2)	0.16	6.6/100	100/53
	FDE (1)	1 (50)			3.414 (0.1288–90.49)	0.483	15.40 (0.607–390.2)	0.16	6.6/100	100/53
B* 55/55:01	MPE (15)	9 (33.3)	07 (11.66)	1 (0.729)	4.929 (1.322–17.33)	0.022	204.0 (27.72–2229)	<0.001	56/79	60/76
B* 40/40:01	Mild-Mode CADRs19	14 (73.68)	14 (46.66)	22 (4.75)	3.200 (0. 885–10.34)	0.080	56.13 (18.42–147.5)	<0.001	50/70	73/53
B* 55/55:01	Mild-Mode CADRs19	9 (47.3)	07 (23.33)	1 (0.21)	2.957 (0.922–10.83)	0.119	415.8 (51.92–454)	<0.001	56/69	47/76
PHT-Over all CADR										
B*51/51:01	CADRs	9 (36)	7 (23.33)	19 (13.8)	1.848 (0.5871–5.905)	0.377	3.493 (1.422–8.652)	0.01	56/58	36/76
B*40/40:01	CADRs	18 (72)	14 (46.66)	22 (16.05)	2.939 (0.9870–8.842)	0.098	13.44 (4.849–33.14)	<0.001	56/69	72/53
B*55/55:01	CADRs	10 (40)	07 (23.33)	1 (0.72)	1.848 (0.5871–5.905)	0.377	76.50 (10.40–841.9)	<0.001	58/60	40/76
B*57/57:01	CADRs	02 (8)	04 (13.33)	6 (4.37)	0.5652 (0.1013–2.643)	0.677	1.899 (0.369–8.216)	0.35	33/53	8/86
B*52/52:01	CADRs	02 (8)	04 (13.33)	11 (8.02)	0.5652 (0.1013–2.643)	0.677	0.996 (0.209–3.973)	>0.99	33/53	8/86
B*07/07:02	CADRs	05 (20)	04 (13.33)	26 (18.9)	1.625 (0.3967–5.800)	0.716	1.067 (0.4067–.909)	>0.99	55/56	20/86
B*15/15:01	CADRs	01 (4)	07 (23.33)	11 (8.02)	0.1369 (0.0117–0.9204)	0.059	0.477(0.042–3.005)	0.69	12.5/48	4/76
B*35/35:01	CADRs	01 (4)	08 (26.66)	22 (16.05)	0.1146 (0.009–0.7273)	0.030	0.2178 (0.020–.370)	0.20	11/47	4/73
CYP2C9*3 carriers										
CYP2C9*3	Severe CADRs (6)	4 (66.6)	2 (7.14)	12 (14.63)	26.00 (2.855–1726)	0.0006	11.69 (2.386–63.63)	0.009	66/92	66/92
CYP2C9*3	DRESS(3)	3 (100)	2 (7.14)	12 (14.63)	74.20 (2.922–1884)	0.002	39.48 (1.920–811.9)	0.004	60/100	100/92
CYP2C9*3	M-Moder. (19)	8 (42.2)	2 (7.14)	12 (14.63)	9.455 (1.628–47.25)	0.008	2.917 (0.9178–0.969)	0.02	80/70	42/92
CYP2C9*3	MPE(15)	5 (33.33)	2 (7.14)	12 (14.63)	6.500 (1.008–35.01)	0.039	5.385 (1.917–13.67)	0.13	71/72	33/92
CYP2C9*3	Overall (25)	12 (48)	2 (7.14)	12 (14.63)	12.92 (2.777–61.46)	0.004	4.242 (1.378–12.89)	0.001	85/66	48/92

SJS, Steven Johnson syndrome; DRESS, drug reactions eosinophilia systemic syndrome; ED, exfoliative dermatitis; MPE, maculo-papular eruption; FDE, fixed drug eruption; LDE, lichenoid drug eruption; AFD, acneiform drug eruption; ^a^ Fisher’s exact test was used for p calculation, ^R1 & R2^ Percentages of HLA B alleles in Tamil population was taken from Leenam Dedhia et al. 2015 [22], and CYP2C9*3 distribution data was taken from Nahar R et al. 2013 [23] After Bonferroni correction, *p*-value 0.008 (<0.05/6—two tailed) were considered significant.

**Table 3:** Pooled data analysis for HLA B*51:01 and PHT-CADRs.

Alleles	Phenotype	Present Study		Literatures		Total after Pooling		Before Pooling		After Pooling		PPV/NPV
		Case	Control	Case	Control	Case	Control	OR (95%CI)	*p* Value	OR (95%CI)	*p* Value	
HLA B*51:01 ^R1^	SCARs	6/6	7/30	4/15	8/100	11/21	15/130	40.73 (2.045–811.3)	0.0009	6.273 (2.24–16.69)	0.0008	46/100 ^a^ 47/8 8^b^
	MM	3/19	7/30	9/35	8/100	12/54	15/130	0.6161 (0.1563–2.603)	0.71	2.190 (0.939–4.97)	0.07	30/58 ^a^ 44/73 ^b^
	Overall CADRs	9/25	7/30	13/50	8/100	22/75	15/130	0.848 (0.5871–0.377)	0.37	2.323 (1.122–5.899)	0.03	36/76 ^a^ 59/68 ^b^

^R1^ Percentage of HLA B*51:01 in PHT-CADRs and tolerant control data was taken from Ihtisham et al. (2019), ^a^ Positive and negative predictive value before pooling, ^b^ Positive and negative predictive value after pooling; SCARs, severe cutaneous adverse drug reactions; OR, odds ratio; CI, confidence interval; MM reac, mild-moderate reactions that includes: MPE, maculo-papular Exanthema; FDE, fixed drug eruption; LDE, lichenoid drug eruption; AFDE, acneiform drug eruption.

**Table 4:** Multivariate binary logistic regression analysis of PHT-CADRs and tolerant-control.

Predictor Variables	β	SE	OR	95% CI	*p* Value
PHT- All types of CADRs/tolerant Control					
CYP2C9*3	2.48	0.83	12	2.759–84.87	0.003
HLA B*51:01	0.48	0.67	1.61	0.429–6.104	0.47
PHT-SCARs/tolerant Control					
CYP2C9*3	2.52	1.22	12.4	1.138–136.2	0.003
HLA B*51:01	2.04	1.45	7.70	0.447–133.0	0.16
HLA B*40:01	−0.33	1.34	0.71	0.52–9.895	0.80
*MPE*/tolerant Control					
HLA B*55:01	1.39	0.66	4.04	1.125–15.67	0.03
HLAB*40:01	0.39	0.78	1.47	0.318–6.873	0.61
CYP2C9*3	1.57	1.05	4.89	0.617–37.79	0.13

β regression coefficient, SE, standard error; OR, odds ratio; CI, confidence interval;MPE, maculo-papularexanthema.

## Supplementary Materials

The following are available online at https://www.mdpi.com/article/10.3390/jpm11080737/s1, Figure S1: Frequently observed HLA B alleles among PHT-CADRs and PHT Tolerant; Table S1 Clinical characteristics and HLA-B and CYP2C9*3 genotyping of the patients with PHT-CADRs.

## Abbreviations

ALDEN	Algorithm of drug causality for epidermal necrolysis
AEDs	Anti-epileptic drugs
CBZ	Carbamazepine
PHT	Phenytoin
DRESS	Drug reaction with eosinophilia and systemic symptoms
HLA	Human leukocyte antigen
MPE	Maculopapular exanthema
NPV	Negative predictive values
SCARs	Severe cutaneous adverse drug reactions
ED	Exfoliative dermatitis
AFDE	Acneiform drug eruption
FDE	Fixed drug eruption
LDE	Lichenoid drug eruption
CADRs	Cutaneous adverse drug reactions
VPA	Valproic acid
TEN	Toxic epidermal necrolysis
