# Supplementary material for: Association of HLA-B*51:01, HLA-B*55:01, CYP2C9*3, and Phenytoin-Induced Cutaneous Adverse Drug Reactions in the South Indian Tamil Population
Source: J Pers Med. 2021 Jul 28;11(8):737. doi: 10.3390/jpm11080737 (PMC8400937; doi:10.3390/jpm11080737)

Supplementary Table S1 Clinical characteristics and HLA-B and CYP2C9*3 genotyping of the patients with PHT-CADRs

S.No	Sex	Age	Phenotype	Onset (days)	Skin/MM/Systemic	HLA-B genotyping		*CYP2C9*3* genotyping	Causality Score	
						Allele 1	Allele 2		Naranjo's	Others
1	F	65	ED	26	Dry scaling/Eyes Oral /Liver & hemat.abn.	*B*51:01*	*B*40:01*	*CYP2C9*3*	*7*	
2	M	64	DRESS	09	Pap. Rash/ Eyes, Oral &genital/ liver & hemat.abn./LA	*B*51:01*	*B*40:01*	*CYP2C9*3*	*7*	7 (RegiSCAR)
3	M	03	DRESS	15	Pap. Rash/ Eyes, Oral &genital/ Liver & hemat.abn./LA	*B*51:01*	*B*40:01*	*CYP2C9*3*	*7*	6 (RegiSCAR)
4	M	09	DRESS	21	Pap. Rash/ Eyes, Oral, genital& anogenital/ Liver & hemat.abn./LA	*B*51:01*	*B*40:01*	*CYP2C9*3*	*7*	7 (RegiSCAR)
5	F	42	SJS	15	Erythema/T.L/ Eyes, Oral, genital	*B*07:02*	*B*51:01*	*CYP2C9*1*	*7*	7 (ALDEN)
6	M	39	SJS	9	Erythema/T.L/ Eyes, Oral, genital	*B*35:01*	*B*51:01*	*CYP2C9*1*	*7*	8 (ALDEN)
7	M	35	LDE	42	Sym. Papules/Oral	*B*51:01*	*B*40:01*	*CYP2C9*3*	*7*	-
8	F	18	AFED	28	Red acne pustules /-/-	*B*52:01*	*B*40:01*	*CYP2C9*1*	*6*	-
9	F	42	AFED	21	Papules –face& neck/-/-	*B*51:01*	*B*40:01*	*CYP2C9*3*	*6*	-
10	M	60	FDE	35	Pigmented single target lesions at the lower back/-	*B*07:01;*	*B*40:01*	*CYP2C9*3*	*7*	-
11	M	23	MPE	21	Red spot rashes and few lesions/-/-	*B*55:01*	*B*40:01*	*CYP2C9*1*	*6*	-
12	M	28	MPE	35	Red spot rashes and few lesions/-/-	*B*55:01*	*B*40:01*	*CYP2C9*3*	*7*	-
13	M	56	MPE	42	Itching/Rashes/	*B*55:01*	*B*40:01*	*CYP2C9*1*	*7*	-
14	F	29	MPE	28	Itching/ macular Rashes/ -/-	*B*55:01*	*B*40:0;*	*CYP2C9*3*	*5*	-
15	M	49	MPE	7	macular Rashes /Itching/-/-	*B*40:01*	*B*40:01*	*CYP2C9*1*	*6*	-
16	F	50	MPE	35	macular Rashes/Itching/-/-	*B*07: 02*	*B*07:02*	*CYP2C9*1*	*5*	-
17	M	40	MPE	21	Itching /Exanthema/Oral/-	*B*07 :02*	*B*07:02*	*CYP2C9*1*	*6*	-
18	F	22	MPE	28	Itching /Red macular patches/-/-	*B*55:01*	*B*40:01*	*CYP2C9*3*	*7*	-
19	M	65	MPE	21	Red spot rashes/-/-	*B*40:01*	*B*40:01*	*CYP2C9*3*	*6*	-
20	F	38	MPE	21	Pruritus/Rashes/	*B*40:01*	*B*15:01*	*CYP2C9*1*	*5*	-
21	F	67	MPE	14	Itching/Exanthema/Red eyes, FE/-	*B*40:01*	*B*57:01*	*CYP2C9*1*	*7*	-
22	M	62	MPE	18	Exanthema/ few target lesions	*B*55:01*	*B*40:01*	*CYP2C9*1*	*7*	-
23	M	42	MPE	20	Exanthema/FE	*B*51:01*	*B*55:01*	*CYP2C9*1*	*7*	-
24	F	29	MPE	07	Red spot rashes/-/-	*B*55:01*	*B*57:01*	*CYP2C9*3*	*6*	-
25	F	35	MPE	21	Red spot rashes, Target lesions/-/-	*B*55:01*	*B*55:01*	*CYP2C9*1*	*6*	-

Supplementary Figure S1: Frequently observed HLA B alleles among PHT-CADRs and PHT-Tolerant.

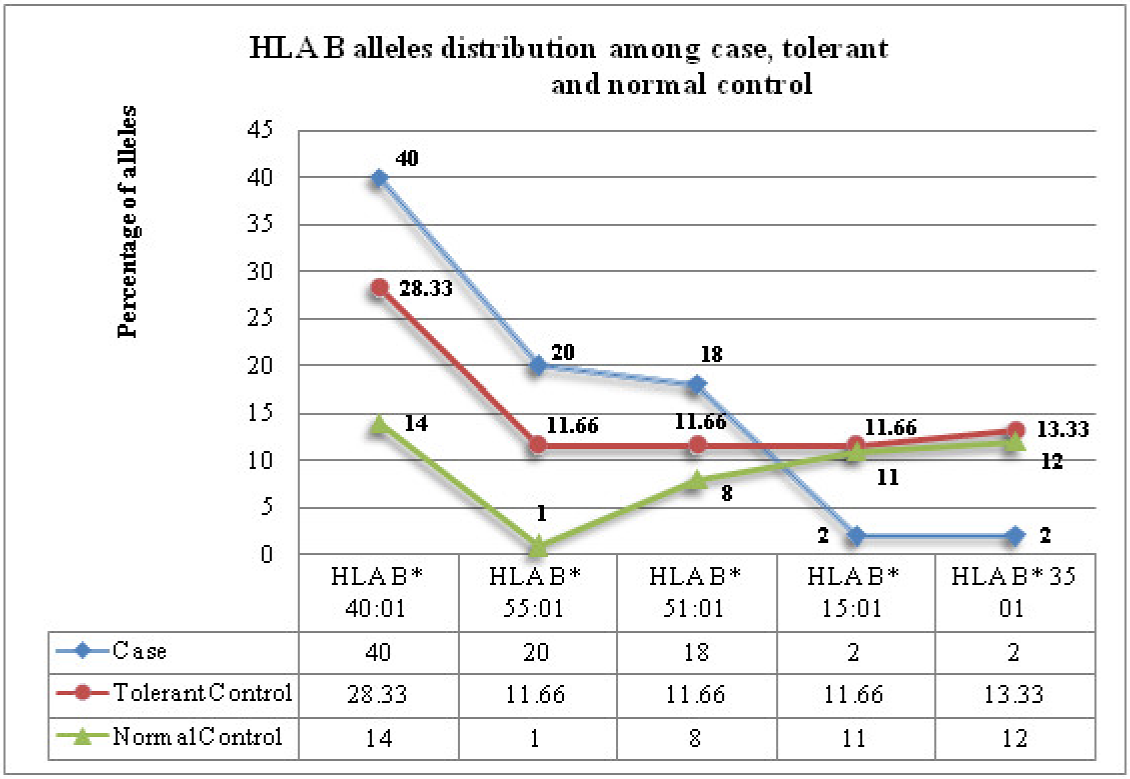

Supplement: Supplementary file 1 [file jpm-11-00737-s001.zip › jpm-1302744-supplementary.pdf]
